# A Complete Hand-book of Treatment

**Published:** 1888-01

**Authors:** 


### BOOK NOTICES.

A COMPLETE HAND-BOOK OF TREATMENT, arranged as an Alphabetical Index of Diseases, by William Aitken, M. D., edited, with notes, etc., by A. D. Rothwell, M. D., of New York. Pp. 444; price, $2.75. New York: E. B. Treat & Co., 1887.

This hand-book is eminently a book of reference, not a treatise upon diseases. The utility and the value of these books have been amply tested, and their popularity is great. The name of Dr. Aitken is a sufficient guarantee for accuracy and reliability. It is of course allopathic in theory and practice, and hence only useful for ready reference in cases where help is quickly needed in diagnosis, etc.

